# Oral ulcers in children- a clinical narrative overview

**DOI:** 10.1186/s13052-021-01097-2

**Published:** 2021-06-30

**Authors:** Corinne Légeret, Raoul Furlano

**Affiliations:** 1 University Children’s Hospital of Basel, Spitalstrasse 33, 4056 Basel, Switzerland; 2 University Children’s Hospital Basel, Spitalstrasse 31, 4055 Basel, Switzerland

**Keywords:** Oral ulcers, Stomatitis, Children, Overview

## Abstract

The prevalence of oral ulcers in children is reported to be 9%, however diagnosis of oral lesions can be challenging, being an unspecific symptom of several diseases. Differential diagnosis can range from classic infectious disease of childhood (e.g. herpangina, hand-foot-and-mouth-disease) over nutritional deficiencies, gastrointestinal disorders, inflammations (e.g. pemphigus vulgaris, lichen planus, mucous membrane pemphigoid) to side effects of medications (Stevens-Johnson Syndrome) or chronic dieseases (e.g. sarcoidosis, systemic Lupus erythematodes, familial Mediterrenean fever). Therefore, children with oral ulcers are treated by many different specialists such as dentists, family doctors, paediatricians, rheumatologists, haematologists, gastroenterologists and otorhinolaryngologists.

A systematic literature search and a narrative literature review about the potential 48 diseases connected to oral ulcers were performed. According to the duration of symptoms and size of the lesions, a tabular overview was created to support the clinician in making a correct diagnosis, additionally different treatment options are presented.

## What is known

- 9% of all children are affected from oral ulcers
- many different specialists see and treat children with ulcers, which might be the reason that no uniform clinical guidelines exist

## What is new

- we have created a narrative review of all differential diagnosis of oral ulcers in children
- a tabular overview, according to duration and size of ulcers, is provided to support clinicians in making a correct diagnosis

## Background

The term ‘aphthous’ is derived from the Greek word *aphthi*, which is translated with ‘to set on fire’ or ‘to inflame’ and is thought to have been first mentioned by the philosopher Hippocrates to describe the pain associated with a frequent disorder of the mouth during this time (equals aphthous stomatitis [1]). The term ‘aphthous stomatitis’ has been used interchangeably with ‘aphthous ulcers’, but at present, the term aphthous stomatitis is preferred [2]. It is a common disease, affecting about 20% of the general population, in children the estimated prevalence is 9% [2]. Due to the high prevalence medical and dental professionals are repeatedly confronted with paediatric patients complaining about oral ulcers, but the diversity of causative factors can make a diagnosis challenging. Causes can range from infections to allergies, nutritional deficiencies, autoinflammation, genetics or can be drug induced, therefore children with oral ulcers are often treated by general paediatricians, dentists, rheumatologist, allergologists and many more specialists.

Although oral ulcers are common in childhood, there is only sparse literature published describing the diagnostic approach. Our aim was to introduce a narrative review and create a decision flowchart for diagnosing children with oral ulcers, supporting clinicians in the diagnostic pathway.

## Methods and results

A systematic literature search was carried out using PubMed Science databases between January 1965 and December 2020 using the following MeSH terms: ‘Oral ulcers’, ‘oral stomatitis’ and ‘children’.

A total of 199 articles were published since 1965 including case reports, cross-sectional studies, retrospective cohort studies and case series. Eighty one articles were excluded because full text was not available anymore or being duplicates. Studies were restricted to humans and conference abstracts, and non-English literature was excluded.

### Classification

An ulcer is defined as a complete breach of the epithelium, which becomes covered with a fibrin slough and appears as a white lesion surrounded by erythema. If a mucosal lesion lasts over 14 days, it is considered chronic, otherwise it is regarded as an acute ulcer [3]. Recurrent ulcers are defined as similar episodes with intermittent healing [4] and are described as recurrent aphthous stomatitis (RAS). RAS are subdivided into minor, major and herpetiform aphthae [5]: Minor aphthae represent the most common variety of RAS (80–85%), which vary between 3 and 10 mm in size and typically involve nonkeratinized oral mucosa (lips, cheeks, floor of the mouth, ventral and lateral surface of tongue). During an episode of minor aphthae, a maximum of 5 ulcers occur and last for 10–14 days before they heal without scarring.

Major aphthae are larger (exceeding 10 mm) and deeper as minor aphthae, they persist longer (over 6 weeks) and can leave a scar after healing. Clinically they impress with substantial pain, fever, dysphagia and malaise.

10% of RAS classify as herpetiform aphthae, the least common type. It is defined as multiple recurrent crops of at least 10 (up to 100 ulcers may be present at the same time) small ulcers of 2-3 mm but may fuse into larger ulcers (as seen in viral infections, thus the name).

Furthermore, oral ulcers are classified as solitary and multiple ulcerative lesions [6].

### Local and systemic etiologies of oral ulcers

**Fig. 1:**
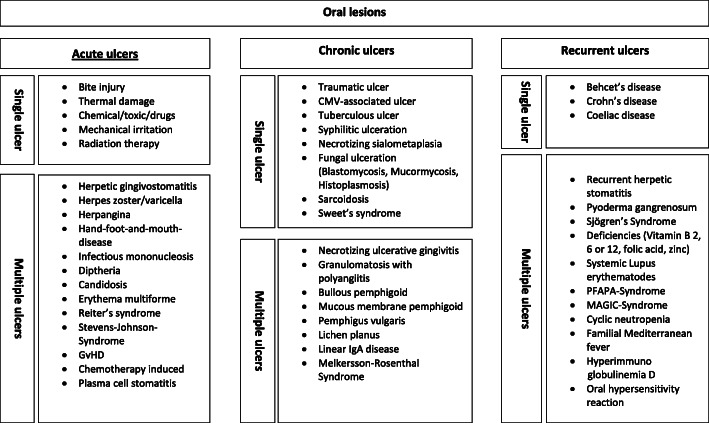
Overview: Oral lesions

A detailed history of the patient is essential to identify whether there are other symptoms present indicating a possible underlying infection, a background of an autoimmune process, immunosuppression or the involvement of the gastrointestinal tract, or whether it is just a local problem. Figure 1 provides a summary of all potential differential diagnosis of oral ulcers, whilst Table 1 gives a tabular overview of diseases according to the timely occurrence and size of the ulcers.

**Table 1:** Clinical characteristics

Disease	Number of ulcers	Location and description of ulcers	Other hints and symptoms
Traumatic ulcer	Depends on trauma	- Can affect all sites of oral cavity - Raised ulcers with reddish borders, necrotic pseudomembrane.	
Herpetic gingivostomatitis	Multiple	- Keratinized and nonkeratinized mucosa - Superficial fluid-filled vesicles, form into ulcers with scalloped borders and erythematous halo	6 months to 5 years Pyrexia, anorexia, submandibular lymphadenitis, dysphagia.
Herpangina	Multiple	- Oropharynx and soft palate - Small vesicular lesions on the.	< 5 years Pyrexia, headache, drooling, peaks in the summer.
Hand-foot-and-mouth-disease	Multiple	- Affecting front of the mouth (tongue, buccal mucosa, hard palate) - Small blisters	< 5 years Pyrexia, rash on hands and feet.
Infectious Mononucleosis	Multiple	- Affects the lateral border of the tongue - Hairy leukoplakia: White, hyperkeratotic lesion with a flat surface	Asymptomatic in early childhood, teenagers and immune-compromised. Pyrexia, halitosis, cervical lymphadenopathy, tonsillitis, hepatosplenomegaly.
Diptheria	Multiple	- Upper respiratory tract, -Formation of pseudomembrane.	Pyrexia, lymphadenopathy. Lack of vaccination.
Fungal infection	Multiple	- Buccal mucosa - White patches, ‘plaques’	Any age in immune-compromised.
Erythema multiforme	Multiple	- Lips, tongue and buccal mucosa - Large and confluent lesions, bullae and ulcerations with irregular border, inflammatory halo and pseudomembrane	Cutaneous ‘target’ lesions. Acute and self-limiting.
Reiter’s syndrome	Multiple	- On the palate, tongue, lips - Superficial, small ulcers or opaque vesicles	A history of bacterial gastroenteritis. Urethritis, conjunctivitis, arthritis. Association with HLA-B27.
Stevens-Johnson-Syndrome	Multiple	- Lips, buccal mucosa, tongue - Large and confluent lesions, same oral presentation as in Erythema multiforme.	Prodromal illness, severe erythematous papules, bullae and skin erosions, ‘target’ lesions (same as in erythema multiforme) mainly on the trunk. Potentially life-threatening, high fever, signs of systemic toxicity.
GvHD	Multiple	- Any site intraorally as well as lips can be involved. - Ulcers can be shallow or deep and confluent.	Skin, gastrointestinal tract, liver, joints might be affected as well.
Plasma cell stomatitis	Multiple	Gingiva presents with desquamative gingivitis.	Angular cheilitis, fissured lips, epithelial desquamation, self-limiting.
CMV-associated ulcer	Single	- Hard and soft palate - Shallow ulcer with rolled margins and yellow slough.	Pyrexia, myalgia, hepatitis, lymphadenopathy, mainly in immunocompromised children.
Tuberculous ulcer	Single	- Gingiva, mucobuccal folds - Single lesion with undermined borders.	Submandibular lymphadenopathy.
Syphilitic ulceration	Single	- Punched-out lesion, 2-3 cm in diameter, covered by a yellow serous discharge.	Lesion lasts for 2–4 weeks, cervical lymphadenopathy.
Necrotizing sialometaplasia	Multiple	- Affecting papillary and marginal gingivae - Crater like ulcer	Fever, halitosis, bleeding gingiva, necrosis of interdental papilla.
Sarcoidosis	Single	Superficial ulcer, sometimes combined with nodules.	Swelling of buccal mucosa.
Necrotizing ulcerative gingivitis	Multiple	- Gingiva - Crater like ulcers with interdental bleeding.	Fever, malaise. Risk factors: Smoking, trauma, preexisting gingivitis or immunosuppression.
Granulomatosis with polyangiitis	Multiple	Strawberry-like gingivitis.	Petechial haemorrhages in the gingivae, hyperplastic gingival lesions.
Bullous pemphigoid	Multiple	- Gingiva - Small vesicles	Desquamative gingivitis.
Mucous membrane pemphigoid	Multiple	- Gingiva and palate - Small blisters	Oesophageal and nasal mucosa can be affected. Bleeding into bullae.
Pemphigus vulgaris	Multiple	- Buccal mucosa and gingivae - Blisters, whose ruptures form erosions	Positive Nikolsky sign, desquamative gingivitis.
Lichen planus	Multiple	- affects buccal mucosa and gingiva - Reticular, erosive and atrophic forms can affect	Inflammatory dermatosis involving wrists, lower limbs and genital mucosa. Desquamative gingivitis.
Linear IgA disease	Multiple	- Mainly buccal - Many blisters with an erythematous base	Same ulcers can appear anogenital.
Melkersson-Rosenthal Syndrome	Multiple	Small, multiple ulcers.	Oro-facial edema, facial nerve palsy, furrowing of the tongue.
Behcet’s disease	Multiple	- Affecting oral or pharyngeal mucosa - Recurrent ulcers covered with sharp erythematous border	Genital ulcers, skin lesions (erythema nodosum- like), ocular inflammation.
Crohn’s and Coeliac disease	Single	- Buccal mucosa - Tag-like lesions	Cobblestoning and mucogingivitis.
Pyoderma gangrenosum	Single or Multiple	- Affecting the tongue - Large, deep ulcers	Necrotic ulcers with a ragged violaceous border and surrounding erythema affecting any anatomical site.
Deficiencies	Multiple	Multiple small ulcers.	Anorexia. Vitamin B, folic acid and zinc deficiencies can be the reason.
Systemic Lupus erythematodes	Multiple	White plaques, raised keratotic plaques, petechiae, cheilitis.	Butterfly-shaped rash, fatigue, fever, joint pain.
PFAPA-Syndrome	Multiple	- Affecting lips and buccal mucosa, not the tongue - Small ulcers	Pharyngitis, cervical lymphadenopathy, joint pain, fever, high CRP.
Cyclic neutropenia	Multiple	- Any oral mucosa - Multiple small ulcers with erythematous halo	Episodic with concomitant fever, periodontitis, gingival recession.
Familial Mediterranean fever	Multiple	Multiple small ulcers	Family history of FMF; High CRP, ESR and fever during the episode, which lasts 1–4 days. Arthritis, serositis.
Oral hypersensitivity reaction	Multiple	Many different forms, which can affect all sites intraorally.	Swelling of the lips, signs of oral allergy.

Traumas might be the easiest causes to be identified: They may be thermal, chemical or mechanical and arise from direct application of heat, acidity or pressure. Repetitive traumatic ulcers or bite injuries can be observed in children with an Autism Spectrum disorder and intellectual disability [7] or in the context of a seizure. Other causes for repetitive local, traumatic ulcers can be ill-fitting dental braces or aggressive toothbrushing. Patients with a nighttime habit of grinding teeth (bruxism) may also suffer from recurrent ulcers in the cheeks, whilst having other symptoms such as a damaged tooth enamel or a deformation of the tongue. Risk factors for bruxism are excessive anxiety, beverages containing caffeine, mental retardation, hyperactivity and cerebral palsy [8]. Mucosal damage is the most common acute side effect experienced by patients undergoing radiation therapy of the head and neck. Clinically it presents as erythema, mucosal atrophy and ulceration with or without pseudomembranes [9].

Eosinophilic ulcer also known as traumatic ulcerative granuloma, oral traumatic granuloma or traumatic ulcer is a benign, self-limited oral condition which often occurs on the lateral tongue but may also be seen in gingival and other areas of the oral mucosa [10]. It appears as a sharp, punched-out ulcer, often in association with a trauma, particularly in young children when teething.

### Viral/bacterial infections

Herpetic gingivostomatitis is a manifestation of herpes simplex virus type 1 (HSV-1), it is the most common viral cause of oral ulcers and occurs in children from 6 months to 5 years. The initial sign is hyperemia of the oral and perioral mucosa, patients are typically febrile with widespread superficial fluid-filled vesicles which rapidly break down to form a cluster of small ulcers of both keratinized and non-keratinized mucosa. On physical examination, they may appear as flat, yellowish in color and approximately 2-5 mm in size, they are quick to bleed and heal without scarring within 18 days [11]. Approximately a third of those patients will develop recurrent episodes of herpes labialis. Ulcers due to Herpes simplex virus 2 (HSV-2) look similar to those of primary HSV-1 infections and are a consequence of orogenital or vertical (from mother to baby) transmission.

Herpangina affects mainly children under 5 years of age, who present with sudden pharyngeal pain, fever, and multiple ulcers in the posterior pharyngeal wall. It is mainly caused by enteroviruses, particularly coxsackievirus, and presents with epidemic peaks in the summer months [12]. Hand-foot-and-mouth-disease (HFMD) is caused by the same group of virus and clinically presents with multiple oral blisters after a prodromal phase of fever and sore throat. Whilst Herpangina lesions are typically only found in the back of the mouth (palatine arch, soft palate, uvula and tonsil), HFMD blisters occur at the front of the mouth and cause lesions on the soles of feet and the palms. Both diseases are usually mild and self-limiting [13].

The primary infection with Epstein-Barr virus (EBV) is typically characterized by fever, pharyngitis, lymphadenopathy and fatigue, it rarely causes neurological complications, as well [14]. Clinical examination might reveal hepatosplenomegaly and laboratory findings include the presence of atypical lymphocytosis and specific antibodies. Oral hairy leukoplakia is a benign, asymptomatic, white, hyperkeratotic lesion affecting primarily the lateral border of the tongue, unilaterally or bilaterally. Its surface may be flat and vertically corrugated. Factors that determine whether productive EBV replication within the oral epithelium will cause oral hairy leukoplakia include the fitness of local immune responses, the profile of EBV gene expression and local environmental factors [15].

Cytomegalovirus (CMV) infection often remains asymptomatic in healthy children and adults, but clinical symptoms can include fever, myalgia, cervical lymphadenopathy and mild hepatitis. It can cause shallow oral ulceration with rolled margins and a yellow slough or pseudomembrane most prevalent on the hard and soft palate [16]. Human cytomegalovirus-associated oral disease is mostly seen in patients receiving immunosuppression or HIV (human immunodeficiency virus) positive, they may develop recurrent aphthous oral ulceration.

Primary oral tuberculosis lesions are rare, but do occur in children [17], affecting the gingiva and mucobuccal folds, usually presenting with a single lesion associated with enlarged submandibular lymph nodes.

In sexually active teenagers with unclear oral ulcers, one has to consider Syphilis, an infection caused by Treponema pallidum, which often manifests orally first [18]. The clinical features of syphilis are diverse as in the case of a primary infection chancre occurs (punched-out lesion, 2–3 cm in diameter without a red inflammatory border, covered by a yellowish serous discharge and lasting 2–4 weeks) at the site of initial infection. An important characteristic of the syphilitic lesion is the absence of painful symptomatology. Secondary syphilis with diffuse and painless maculopapular cutaneous rash (syphilitic rosette) can also affect the oral cavity: Reddish oval macules and ulcers surrounded by an erythematous halo.

A differential diagnosis for white lesions is diphtheria, a disease caused by Corynebacterium diphtheriae in children with no vaccination records. It initially presents with fever, sore throat and cervical lymphadenopathy. Ulcerae are localized in the upper respiratory tract and induce the formation of an inflammatory pseudomembrane. Unusual sites of infection include buccal mucosa, lips, tongue and hard palate [19]. Rarely cutaneous diphteria can be seen, where ulcerating skin lesions are present, which are covered with a gray membrane.

Necrotising ulcerative gingivitis is a rare disease with an acute, painful and destructive presentation with interdental gingival necrosis and bleeding. It was classically seen among military personnel during World War I, due to poor oral hygiene, stress and malnutrition. Nowadays risk factors are smoking, trauma, preexisting gingivitis or immunosuppression. It is an opportunistic bacterial infection, predominantly associated with spirochetes [20].

### Fungal infections

In the oral cavity superficial and invasive fungal infections are encountered, mainly in immunocompromised patients (e.g. primary immunodeficiencies, HIV, secondary immunodeficiencies caused by medications). Candida infection is unlikely to cause oral ulcerations, however acute pseudomembranous candidiasis or acute atrophic candidiasis may do so [21].

Mycoses to be considered causing oral ulcerations include zygomycosis, aspergillosis, histoplasmosis, blastomycosis and paracoccidiomycosis [22].

### Nutritional deficiency

Recurrent aphthous stomatitis can be caused by deficiency of Vitamin B complex, folic acid [23], iron [24] or zinc [25]. Typically, minor aphthae can be seen.

### Gastrointestinal diseases

As the oral cavity is part of the gastrointestinal tract, recurrent oral ulcers can also be caused by gastrointestinal diseases. Typically, Coeliac disease can present with multiple small ulcers with circumscribed margins and yellow/gray floors [26], additionally the child might complain about abdominal pain, bloating or diarrhea. The same manifestations or angular cheilitis, perioral erythema and labial swelling can be induced by an inflammatory bowel disease (Crohn’s disease, ulcerative colitis or undefined colitis) [27].

### Medications

Non-steroidal anti-inflammatory drugs (NSAIDs), β-Blockers, antibiotics, oral anti-diabetics, antihypertensives, platelet aggregation inhibitors, vasodilators and protease inhibitors have been reported to cause oral ulcers, typically isolated ones, located on the side of the tongue and resistant to usual treatments [28]. In 90% of all cases of acute graft-versus-host-disease (aGVHD) buccal mucosa is also affected [29]. Initially, it starts with nonspecific erythema and ulcerations that can affect nonkeratinized and keratinized oral mucosa and can lead to a complete resolution secondary to conditioning regimen.

Stevens-Johnson Syndrome is an immune-mediated disease commonly triggered by medications (NSAR, sulfonamides, anti-epileptics or antibiotics), but also infectious etiologies, especially Herpes simplex virus and Mycoplasma pneumoniae. After a prodromal illness, the disease phenotype is strongly mucus membrane-predominant [30] and can be associated with significant morbidity. Oral manifestations of the disease present as painful erythematous crusts and erosions with a greyish-white membrane.

### Inflammations

Erythema multiforme (EM) is an autoimmune mucocutaneous disorder with an acute onset without prodromal symptoms and can be divided into 2 subgroups: EM without mucous membrane involvement (EM minor) and EM with mucous membrane involvement (EM major). It is a result of a type IV hypersensitivity immune response to an inciting infectious (Herpes simplex type 1 and 2, Mycoplasma pneumonia) or pharmacologic (NSAR, antiepileptics and antibiotics, especially penicillins and sulfonamides) agents [31]. 70% of patients present oral manifestations [32], initially as edematous macular lesions of the lips and buccal mucosa. Advanced lesions manifest as multiple vesiculobullous lesions which break down to form a pseudomembrane. A subset of patients has repeated episodes of EM, which show a lower response to immunosuppression [32].

Some vesiculobullous diseases such as pemphigus vulgaris, mucous membrane pemphigoid and bullous pemphigoid present as multiple and chronic oral ulcerative lesions.

Pemphigus vulgaris is characterized by the presence of circulating autoantibodies immunoglobulin G against desmoglein 3, which results in loss of cell adhesion (acantholysis) and blister formation whose rupture progresses to form painful erosions [33]. The lesions usually affect the buccal mucosa, gingival involvement might be in the form of desquamative gingivitis, which is a characteristic feature for pemphigus vulgaris. As the lesions extend over a period of weeks until they involve large areas of oral cavity, coexisting candidiasis may mask the typical features of the lesions. Mucous membrane pemphigoid, also known as cicatricial pemphigoid, is also a chronic autoimmune disease, which produces autoantibodies (IgG in 97%, IgA in 27%, IgM in 12%), targeted to certain components of the basal lamina of the epithelium [34]. Blisters, mostly affecting the gingiva, occupy the full thickness of the epithelium. Palate, esophagus or nasal mucosa can also be affected. Bleeding into bullae are a diagnostic feature of mucous membrane pemphigoid, as well a positive Nikolsky sign.

Bullous pemphigoid can cause oral mucosal lesions, which are similar to pemphigus vulgaris and mucous membrane pemphigoid but are smaller and less painful. Histology shows an inflammatory infiltrate dominated by eosinophils and subepidermal blistering. Infantile bullous pemphigoid presents with urticarial plaques and blisters, involving hands and feet [35].

Lichen planus is an inflammatory dermatosis presenting with violaceous, polygonal papules involving wrists, lower limbs, genital and oral mucosa, leaving areas of hyperpigmentations after healing. There are different subtypes with different clinical presentations: reticular, atrophic, erosive/ulcerative, bullous and plaque like [36]. Atrophic or erosive Lichen Planus involving the gingivae results in desquamative gingivitis, which has also been found in Pemphigus vulgaris and Mucous membrane pemphigoid [37].

Linear IgA disease got its name after the discovery of linear deposition of IgA antibodies along the basement membrane zone of the skin and/or mucosae on direct immunofluorescence. Clinically it appears as blisters with a possible ‘string of pearls’ configuration and an erythematous/urticarial base, the preferential sites are the anogenital area, as well as the oral cavity and conjunctiva [38]. Drugs are a possible trigger, but there is also an association with inflammatory bowel disease [39]. Mainly preschool-aged children are affected with a self-healing course.

Pyoderma gangrenosum is a rare neutrophilic dermatosis, characterized by recurrent painful cutaneous ulcers, with well-defined, raised, over-hanging, purple-bluish borders, beginning as small follicular pustules. The precise etiology is unclear, but the most popular theory is an immune etiology, supported by the association with immunologic disorders such as rheumatoid arthritis and IBD and its response to treatment with immunosuppressive therapy. There are no specific serologic markers, therefore diagnosis relies on history and physical examination [40]. The condition can affect any anatomical site, but oral lesions appear to be rare [41].

Necrotizing granulomatous inflammation and small-vessel vasculitis of the respiratory tract and kidney are the typical findings in patients with Granulomatosis with polyangiitis, a systemic inflammatory disease. Other organs may be affected, oral manifestations range from 6 to 13% [42] and typically present as strawberry-like gingivitis (reddish-purple exophytic gingival swellings with petechial haemorrhages) or hyperplastic gingival lesions.

Necrotising sialometaplasia is a benign self-limiting inflammatory lesion occurring commonly in the oral cavity in the sites where the minor salivary gland tissue is present mainly on the palate, labial mucosa and floor of the mouth. There is a sudden onset of the ulcer which increases in size, accompanied by pain which radiates to the ear and pharynx and after 4–12 weeks it undergoes a complete resolution [43]. The lesion can be induced as a sequel to surgical procedures, recurrent vomiting, or tobacco consumption leading to an injury of the blood vessels that initially produce ischaemic changes, and then an infarction of salivary glands. The healing process induces short-term metaplasia and changes in ductal architecture.

The etiology of Behcet’s disease is still widely unknown but it appears to be an interaction between the genetic background (HLA-B51, −A26, −B15, −B27 and -B56) and the innate immune system (hyperactivity of neutrophils) which results in a systemic inflammation with recurrent fevers, mucocutaneous lesions, joint, eye, neurologic, vascular and gastrointestinal involvement. Oral manifestations are discrete, round ulcers with a yellow-gray pseudomembranous base and a red halo, affecting lips, tongue, cheeks and palate, disappearing without scarring [44].. The international classifications criteria [45] for Behcet’s disease require recurrent oral aphtosis (at least 3 times in a year, seen by a physician) plus at least two other presentations (genital ulcer, skin lesions, ocular lesions or positive pathergy test). The course is recurrent and unpredictable and often remains active with new symptoms appearing in children.

A differential diagnosis for oral and genital ulcers is the MAGIC (‘Mouth and genital ulcers with inflamed cartilage’) syndrome, if an inflamed cartilage co-exists [46].

Systemic Lupus Erythematosus is a chronic autoimmune disease which can involve any organ system (kidney, gastrointestinal, haematologic, musculoskeletal, pulmonary, cardiovascular, neurologic, cutaneous) including the oral cavity [47]. Oral or nasopharyngeal ulcerations are usually painless and not the leading symptoms. Patients frequently recount nonspecific symptoms such as recurrent fever, anorexia, weight loss and arthralgias.

An important differential diagnosis for recurrent episodes of fever and oral ulcers, accompanied by pharyngitis and cervical adenitis is the Periodic Fever, Aphthous Stomatitis, Pharyngitis, Adenitis- syndrome (PFAPA syndrome) [48]. Disease onset is usually before the age of 5 years and generally resolves by adolescence. Proposed contributors to pathogenesis include infection and a cytokine dysfunction, in combination with a strong familial clustering. Aphthous-like oral ulcerations have been reported as one manifestation in several periodic syndromes, such as Periodic fever syndrome, familial Mediterranean fever, Hyperimmunoglobulinaemia D [49] or cyclic neutropenia [50].

Patients, who present mainly with a dry mouth, Sjogrens syndrome has to be considered, especially when hyposalivation co-exists. It is an uncommon autoimmune condition in the paediatric age group, but can also present with recurrent parotid swelling, unlike in adults [51]. Primary Sjogrens syndrome is not associated with any other illness but can secondly develop in the presence of autoimmune disorders such as rheumatoid arthritis, lupus, scleroderma or polymyositis.

Sweet Syndrome is a febrile neutrophilic dermatosis, characterized by the onset of painful erythematous-violaceous lesions that may be located mainly on limps, trunk, face and neck. Mucosal involvement of the mouth, appearing as oral ulcer, is uncommon [52].

Reiter’s Syndrome, also known as reactive arthritis, is the classic triad of conjunctivitis, urethritis and arthritis occurring after an infection, particularly those in the urogenital or gastrointestinal tract. Dermatologic manifestations are common, including oral lesions, nail changes and ulcerative vulvitis [53]. Further, a differential diagnosis for oral ulcers is Sarcoidosis, a chronic systemic disorder (affects lungs, lymph nodes, skin, eyes etc.) of unknown etiology, which can also affect the buccal mucosa and gingiva [54].

Melkerrson-Rosenthal Syndrome is a rare disorder of unknown aetiology and characterized by the triad of oro-facial edema, facial nerve palsy and furrowing of the tongue. Onset in children is common between 7 and 12 years of age and it can be associated with oral ulcers [55].

#### Treatment options for ulcers

When oral ulcers are secondary to an underlying disease (inflammatory bowel disease, nutritional deficiency etc.) it is recommended to treat the primary disease in order to improve the oral aphtae.

Topical treatment is aimed at prevention of superinfection, analgesia, decreasing inflammation and treating active ulcers. Chlorhexidine has a proven activity against enveloped viruses (HSV, CMV, Influenza, RSV) [56], topical antibiotics (doxycycline, minocycline) are also effective. Bioadhesive pastes with benzocaine 20% achieve pain relief by creating a protective coat, Lidoaine (5% crème or 10% spray) have a temporary analgesic effect, anti-inflammatory properties of diclofenac (3% with hyaluronic acid 2.5%) have also shown to be effective. Topical steroids (betamethasone, fluticasone as spray or mouthwash) are successful in treating active ulcers, and can also be used long term, but only in combination with antifungals to reduce the risk of oral candidiasis [57].

If local treatment is not enough, oral prednisone is used as first line systemic therapy, alternatively Montelukast can be used. Colchicine, Dapsone or Thalidomide are also effective immunomodulators [57]. Ascorbic acid can be added to topical treatment [58].

## Summary

The range of differential diagnosis for oral ulcers in children is enormously wide. Although it is a common symptom, an accurate history, as well as a proper physical examination is of highest importance in order to be able to make a diagnosis. Ulcers can be classified by their size and the duration of symptoms, which can give further hints for the cause of the ulcer. As many different specialists (dentists, gastroenterologists, haematologists, family doctors, paediatricians) treat children with oral ulcers, general recommendations about treatment/diagnosis of children with oral ulcers do not yet exist, therefore an attempt was made to provide a clinical overview for clinicians.

## Data Availability

Available upon request.

## Abbreviations

aGvHD: acute Graft versus Host disease
EBV: Ebstein-Barr virus
HFMD: Hand-foot-and-mouth-disease
HSV: Herpes simplex virus
RAS: Recurrent aphthous stomatitis
